# Association of HER2, p53, and lymphovascular space invasion with lymph node metastasis in endometrial cancer: a retrospective cohort study

**DOI:** 10.3389/fonc.2026.1974528

**Published:** 2026-10-12

**Authors:** Xuqing Cai, Yuanyuan Zhu, Siying Wang, Lingling Gao, Dan Lu

**Affiliations:** 1 Faculty of Medicine, Yangzhou University, Yangzhou, Jiangsu, China; 2 Department of Obstetrics and Gynecology, Northern Jiangsu People’s Hospital Affiliated to Yangzhou University, Yangzhou, Jiangsu, China; 3 Department of Obstetrics and Gynecology, Northern Jiangsu People’s Hospital, Yangzhou, Jiangsu, China

**Keywords:** associated factors, endometrial carcinoma, HER2, lymph node metastasis, lymphovascular space invasion, p53

## Abstract

**Background:**

The assessment of lymph node metastasis (LNM) in endometrial carcinoma is critical for optimizing the extent of surgical resection and formulating individualized therapies. This study aimed to investigate the associated factors of the LNM in endometrial carcinoma.

**Methods:**

438 patients with endometrial carcinoma who underwent primary surgical staging between June 2014 and June 2025 were retrospectively analyzed. Clinicopathological data and immunohistochemical expression profiles of ER, PR, HER2, p53, Ki67, ERCC1, and GST-π were systematically collected. Univariate and multivariate logistic regression analyses were performed to identify independent associated factors for LNM.

**Results:**

The overall incidence of LNM in the study was 10.27% (45/438). Univariate analysis revealed that menopausal status, histological grade, Bokhman pathological subtype, depth of myometrial invasion, lymphovascular space invasion (LVSI), and the expression levels of ER, PR, Ki67, p53, and HER2 were significantly associated with LNM (all P < 0.05). Subsequent multivariate analysis identified each one-category increase in HER2 staining level was associated with higher odds of LNM (OR 3.859, 95% CI 2.314–6.436, P < 0.001), as was each one-category increase in p53 staining level (OR 1.932, 95% CI 1.212–3.082, P = 0.006). The presence of LVSI was also an independent associated factor for LNM.

**Conclusions:**

Higher p53 and HER2 staining levels and the presence of LVSI are independently associated with LNM in endometrial carcinoma.

## Introduction

Endometrial carcinoma is one of the most prevalent malignancies of the female reproductive system. In recent years, its incidence has demonstrated a continuous upward trend, positioning endometrial carcinoma as a critical disease that severely threatens women’s health (1). Although the majority of patients are diagnosed at an early stage due to typical presentation with abnormal uterine bleeding, approximately 10% to 15% of patients present with regional lymph node metastasis (LNM) or high-risk pathological features at the time of diagnosis (2). These patients are highly susceptible to postoperative recurrence and distant metastasis, experiencing a decline in 5-year survival rates. Consequently, accurately and effectively evaluating LNM risk pre- and intraoperatively to formulate optimal, individualized treatment strategies remains a focal point and primary challenge in gynecological oncology.

LNM is not only a core indicator for the International Federation of Gynecology and Obstetrics (FIGO) surgical-pathological staging of endometrial carcinoma, but it also serves as a crucial determinant for adjuvant chemoradiotherapy and long-term survival prognosis (3). Systematic pelvic and para-aortic lymphadenectomy has been considered the standard surgical procedure for obtaining accurate pathological staging and eradicating micro-metastases. However, multiple large-scale prospective clinical trials have demonstrated that for patients with low-risk endometrial carcinoma, systematic LND does not significantly improve overall survival or progression-free survival (4). Conversely, it significantly increases operative time and blood loss, and introduces severe long-term postoperative complications, such as lymphoceles, severe lower extremity lymphedema, and nerve injury, which substantially compromise patients’ quality of life (5).

Prior investigations have established that traditional clinicopathological factors, including histological grade, pathological subtype, FIGO stage, depth of myometrial invasion, and LVSI are intimately associated with LNM in endometrial carcinoma (6, 7). However, relying solely on these conventional parameters falls short of meeting the modern clinical requirements for precise risk stratification. With accumulating insights from molecular pathology, the profound molecular heterogeneity of endometrial carcinoma has become a primary focus, and the integration of molecular classification schemes has begun to reshape clinical risk assessment and management protocols (8). Human epidermal growth factor receptor 2 (HER2), a prominent proto-oncogenic protein, promotes tumor cell proliferation and invasion. Its expression status has been tightly linked to high-risk histological subtypes, molecular profiles, and adverse clinical outcomes in endometrial carcinoma (9). Similarly, abnormal p53 expression typically signals a loss of tumor suppressor function and enhanced malignant biological behavior. Besides, TP53-mutated endometrial carcinoma is characterized by high clinical aggressiveness and poor prognosis (10). Furthermore, Ki67 is widely utilized as a standard indicator of cellular proliferative activity. Beyond these markers, hormone receptors, such as estrogen receptor (ER) and progesterone receptor (PR), alongside drug-resistance-associated molecules like ERCC1 and GST-π, are also implicated in endometrial carcinoma pathogenesis and therapeutic response. However, the precise quantitative relationship between the expression abundance of these markers and regional LNM remains inconsistently reported. Against this backdrop, this study retrospectively analyzed detailed clinicopathological data alongside routine immunohistochemical (IHC) markers (ER, PR, HER2, p53, Ki67, ERCC1, GST-π) from endometrial carcinoma patients. We aimed to identify postoperative pathological factors independently associated with LNM and to provide a basis for future studies investigating whether selected biomarkers can be assessed in preoperative biopsy specimens for clinically useful risk prediction.

## Methods

### Study design and population

Patients diagnosed with endometrial carcinoma who were admitted to and underwent primary surgical treatment at Northern Jiangsu People’s Hospital from June 2014 to June 2025 were collected. Comprehensive clinical and pathological data were systematically extracted from the hospital’s electronic medical record and pathology databases. The study protocol was thoroughly reviewed and approved by the Institutional Review Board (IRB) and Ethics Committee of Northern Jiangsu People’s Hospital (Approval Nos.2023ky114 and 2025ky370). All procedures performed in studies involving human participants were strictly in accordance with the ethical standards of the institutional research committee and with the Helsinki Declaration. Given the retrospective nature of the study and the use of anonymized clinical data, the requirement for written informed consent was formally waived by the ethics committee.

Patients were rigorously screened based on the following inclusion criteria: Primary endometrial carcinoma unambiguously confirmed by postoperative histopathological examination; Patients didn’t receive any neoadjuvant interventions such as radiotherapy, systemic chemotherapy, or hormonal therapy prior to surgery; The standard surgical procedure comprised total hysterectomy and bilateral salpingo-oophorectomy, combined with pelvic lymph node dissection, with or without para-aortic lymph node dissection; Availability of comprehensive and intact medical records, including detailed baseline clinical characteristics, postoperative pathology reports, and complete IHC staining profiles.

To minimize potential confounding variables, patients meeting any of the following exclusion criteria were excluded from the final analysis: A history of or receipt of any prior antitumor therapies (radiation, chemotherapy, or endocrine therapy), which could significantly alter the tumor’s native molecular expression and pathological profile; Incomplete, uninterpretable, or missing postoperative IHC detection results; Presence of concurrent other primary malignancies or a prior history of other cancers; Severe underlying systemic comorbidities, including but not limited to severe cardiac, pulmonary, hepatic, or renal failure, which could independently compromise surgical safety and clinical outcomes.

### Diagnostic criteria, staging, and pathological classification

All surgically resected tumor specimens were rigorously processed, stained with hematoxylin and eosin, and independently reviewed by at least two senior, board-certified gynecological pathologists to ensure diagnostic accuracy and consensus. The staging of the disease was determined postoperatively according to the 2009 FIGO surgical-pathological staging system for endometrial carcinoma because during the study period from 2014 to 2025, FIGO stage was recorded according to the 2009 FIGO staging system in Chinese clinical teaching and practice. Tumor differentiation was systematically evaluated utilizing the standard FIGO three-tier grading system. The tumors were stratified as Well-differentiated (G1), Moderately differentiated (G2) and poorly differentiated (G3). Endometrial carcinomas were additionally categorized according to the historical Bokhman Type I/Type II clinicopathological model. Type I tumors were defined predominantly as endometrioid carcinomas, generally characterized by lower histological grade and an estrogen-related clinicopathological profile. Type II tumors were defined predominantly as non-endometrioid carcinomas, particularly serous and clear cell carcinomas, which are generally characterized by higher-grade histology and a less clearly estrogen-related profile.

### Data collection

Detailed demographic and clinicopathological data were systematically extracted from the patients’ medical records and pathology reports. The specific variables collected included age at diagnosis, menopausal status, pathological classification (Type I or Type II), tumor histological grade, FIGO stage, depth of myometrial invasion, the presence or absence of LVSI, and the definitive status of regional LNM.

Immunohistochemical staining results were retrospectively obtained from routine pathological records. Because the study period extended from June 2014 to June 2025, immunohistochemical staining was performed during different periods according to the routine protocols of the pathology laboratory in use at the corresponding time. ER, PR, HER2, p53, Ki67, excision repair cross-complementation group 1 (ERCC1), and glutathione S-transferase pi (GST-π) were assessed using a historical semi-quantitative scoring system based on the proportion of positively stained tumor cells. Any discrepancies in interpretation were resolved through a joint consensus review. A standardized semi-quantitative scoring criteria was applied to evaluate the immunoreactivity of the markers, strictly based on the percentage of positively stained tumor cells relative to the total tumor cell population in the evaluated fields. The expression intensity was stratified into four distinct tiers: Negative (-): 0% positively stained cells; Weakly positive (+): <30% positively stained cell; Moderately positive (++): 30% to 70% positively stained cells; Strongly positive (+++): 70% positively stained cells. For statistical analysis, these four-category staining results were coded as ordinal variables (0, 1, 2, and 3, corresponding to −, +, ++, and +++, respectively). These categories represent semi-quantitative levels of immunohistochemical staining in the historical pathology records and were used as ordinal measures of expression. They were not used to define p53-abnormal molecular classification or HER2-positive disease according to contemporary endometrial cancer-specific criteria.

### Statistical analysis

All statistical data management and analyses were conducted utilizing Stata statistical software, version 15.0 (StataCorp LLC, College Station, TX, USA). Descriptive statistics were employed to summarize the baseline clinicopathological characteristics and biomarker expression profiles of the study cohort. Categorical and ordinal variables were presented as absolute frequencies and relative percentages.Pearson’s Chi-square test or Fisher’s exact test was utilized for nominal categorical variables (such as menopausal status and pathological subtype). The Mann-Whitney U test was specifically applied for ordinal categorical variables, including the three-tier FIGO histological grade and the semi-quantitative IHC intensity scores. For ordinal immunohistochemical variables, staining intensity was coded sequentially as 0, 1, 2, and 3 for 0, 1+, 2+, and 3+, respectively, and entered into the logistic regression models as a single ordinal predictor. To rigorously identify independent associated factors predictive of LNM, variables that demonstrated statistically significant associations with LNM in the univariate analyses were subsequently incorporated into a multivariate binary logistic regression model. For each variable, analyses were based on patients with available data, and the number of non-missing observations was reported in the corresponding tables. Missing observations were not imputed and were excluded from the analysis involving the affected variable. For descriptive and univariate analyses, available observations were used for each variable, and the number of observations therefore varied according to data availability. For the multivariable binary logistic regression analysis, complete-case analysis was performed. Patients with missing values for any variable included in the multivariable model were excluded. The predictive impact of each independent associated factor was quantified and reported as an adjusted odds ratio (OR) alongside its corresponding 95% confidence interval (95% CI). All statistical tests performed were two-sided. A P-value of < 0.05 was established as the threshold to indicate statistical significance.

## Results

### Baseline clinicopathological characteristics

A total of 438 patients with endometrial carcinoma who underwent surgical staging, including lymphadenectomy, were enrolled in this study. The mean age of the overall cohort was 56.37 ± 9.72 years. The study population included –149 postmenopausal women and 289 premenopausal patients, with a mean age at menopause of 50.71 ± 4.89 years. According to the pathological classification, 381 patients had Type I endometrial carcinoma, while 57 patients had Type II endometrial carcinoma. Histological grade data were available for 428 patients, including 61 well-differentiated (G1), 283 moderately differentiated (G2), and 84 poorly differentiated (G3) tumors. Data were missing for 10 patients. Evaluation of the depth of myometrial invasion revealed that 307 patients had superficial invasion (< 1/2 of the myometrium), whereas –131 patients exhibited deep invasion (≥ 1/2 of the myometrium). LVSI status was available for 431 patients, with LVSI present in 55 cases and absent in 376 cases. LVSI data were missing for 7 patients. Based on definitive postoperative pathological examinations, regional LNM occurred in 45 patients, yielding an overall LNM incidence rate of 10.27% (45/438). The remaining 393 patients (89.73%) were confirmed to be negative for LNM.

### Univariate analysis of clinicopathological features and lymph node metastasis

Compared with the non- LNM group, patients in the LNM group demonstrated statistically significant differences in menopausal status, histological grade, pathological subtype, depth of myometrial invasion, and LVSI (**Table 1**). Specifically, poorer histological differentiation was significantly associated with an increased rate of LNM (P = 0.020). The proportion of Type II endometrial carcinoma was markedly elevated in the LNM cohort compared to the non-LNM cohort (P < 0.001). Furthermore, patients presenting with deep myometrial invasion and positive LVSI were significantly more susceptible to developing LNM (both P < 0.001). Collectively, these univariate findings strongly indicate that menopausal status, histological grade, pathological subtype, depth of myometrial invasion, and LVSI are closely correlated with the risk of LNM in patients with endometrial carcinoma.

**Table 1:** Univariate analysis of clinicopathological features and lymph node metastasis.

Clinicopathological features		LNM group (n=45)	Non-LNM group (n=393)	Statistic	P
Menopausal status	Yes	9	140	4.3908	0.036*
	No	36	253		
Histological grade (n=428)	G1	2	59	7.8485	0.020*
	G2	25	258		
	G3	14	70		
FIGO stage	Stage I+II	18	211	3.0330	0.082
	Stage III+IV	27	182		
Pathological subtype	Type I	31	350	14.5103	< 0.001**
	Type II	14	43		
Myometrial invasion	<1/2	19	288	18.5814	< 0.001**
	≥1/2	26	105		
LVSI (n=431)	Yes	16	39	28.0738	< 0.001**
	No	25	351		

Percentages were calculated based on available observations for each variable. Missing observations were not included in the corresponding analysis. Pearson’s chi-square test or Fisher’s exact test was used for categorical variables, as appropriate. The Mann–Whitney U test was used for ordinal variables. χ² denotes the Pearson chi-square statistic, and Z denotes the standardized test statistic from the Mann–Whitney U test. ^**^P<0.01, ^*^P<0.05.

### Association between molecular biomarker expression levels and lymph node metastasis

Mann-Whitney U test was conducted to evaluate the association between the semi-quantitative expression levels (graded from 0 to +++) of various molecular biomarkers (ER, PR, Ki67, p53, HER2, ERCC1, and GST-π) and the presence of LNM. The analysis revealed statistically significant differences in the expression levels of ER, PR, Ki67, p53, and HER2 between patients with and without LNM (all P < 0.05). Specifically, the expression levels of ER (Z = -2.22, P = 0.026) and PR (Z = -2.77, P = 0.006) were significantly lower in the LNM group, indicating an inverse association with the occurrence of metastasis. Conversely, the expression levels of Ki67 (Z = 3.64, P < 0.001), p53 (Z = 5.88, P < 0.001), and HER2 (Z = 7.93, P < 0.001) were markedly elevated in patients with LNM, demonstrating a strong positive association with metastatic progression. In contrast, no statistically significant differences were observed between the LNM and non-LNM cohorts regarding the expression levels of the drug-resistance-associated markers ERCC1 (Z = -0.07, P = 0.947) and GST-π (Z = 0.61, P = 0.539) (**Table 2**).

**Table 2:** Association between molecular biomarker expression levels and lymph node metastasis.

Molecular biomarker		LNM		Z	P	Molecular biomarker		LNM		Z	P
		Yes	No					Yes	No		
ER (n=438)	0	5	27	-2.22	0.026*	HER2 (n=431)	0	7	266	7.93	< 0.001**
	1+	9	56				1+	17	90		
	2+	12	66				2+	17	30		
	3+	19	244				3+	2	2		
PR (n=436)	0	6	33	-2.77	0.006**	ERCC1 (n=396)	0	16	163	-0.07	0.947
	1+	15	59				1+	12	121		
	2+	6	77				2+	5	45		
	3+	18	222				3+	3	31		
p53 (n=430)	0	7	108	5.88	< 0.001**	GST-π (n=428)	0	3	22	0.61	0.539
	1+	12	209				1+	13	123		
	2+	9	46				2+	19	200		
	3+	16	23				3+	8	40		
Ki67 (n=438)	1+	8	181	3.64	< 0.001**						
	2+	21	135								
	3+	16	77								

For ordinal immunohistochemical variables, ORs represent the change in the odds of lymph node metastasis associated with each one-category increase in staining intensity (0, 1+, 2+, and 3+ coded as 0, 1, 2, and 3, respectively) ^**^P<0.01, ^*^P<0.05.

### Multivariate logistic regression analysis of associated factors for lymph node metastasis

To further elucidate the independent predictive factors for LNM in endometrial carcinoma, variables that demonstrated statistical significance in the univariate analysis were incorporated into a multivariate logistic regression model. Menopausal status, histological grade, pathological subtype, depth of myometrial invasion, LVSI, ER, PR, p53, Ki67, and HER2 were included in the model. Among the 438 patients in the overall cohort, 387 patients with complete data for all variables included in the multivariable model were included in the final binary logistic regression analysis. The remaining 51 patients were excluded because of missing values in one or more model variables. Among the 387 patients included in the model, 36 had LNM. In the multivariable logistic regression analysis, LVSI, p53 staining level, and HER2 staining level were independently associated with LNM. Notably, HER2 expression showed the largest estimated association among the ordinal immunohistochemical biomarkers included in the model (OR 3.859, 95% CI 2.314–6.436, P<0.001). This indicates that among each one-category increase in HER2 staining intensity was associated with a 3.859-fold increase in the odds of lymph node metastasis. In the multivariable binary logistic regression analysis, higher p53 expression was independently associated with LNM (OR 1.932, 95% CI 1.212–3.082, P = 0.006). Because p53 expression was analyzed as an ordinal immunohistochemical variable, the OR represents the change in the odds of LNM associated with a one-category increase in p53 expression. These findings describe associations with the semi-quantitative IHC staining levels and should not be interpreted as evidence of p53-abnormal molecular classification or HER2-positive disease. Furthermore, LVSI also demonstrated a significant impact on the occurrence of LNM (Z = 2.14, P = 0.033), with an OR of 1.603 (95% CI: 1.084–6.526) (**Table 3**).

**Table 3:** Multivariate logistic regression analysis of associated factors for lymph node metastasis.

Associated factors	OR	SE	z	P	95%CI
Menopausal status	0.809	0.286	-0.60	0.549	0.404-1.617
Histological grade	1.086	0.313	0.29	0.774	0.618-1.909
Pathological subtype	0.786	0.436	-0.43	0.664	0.265-2.332
Myometrial invasion	2.731	2.042	1.34	0.179	0.631-11.822
LVSI	2.659	1.218	2.14	0.033*	1.084-6.526
ER	0.794	0.245	-0.75	0.455	0.433-1.454
PR	0.837	0.260	-0.57	0.567	0.454-1.540
P53	1.932	0.460	2.77	0.006*	1.212-3.082
Ki67	1.368	0.376	1.14	0.255	0.798-2.346
HER2	3.859	1.007	5.17	0.000*	2.314-6.436

387 patients were included using complete-case analysis, including 36 patients with LNM and 351 patients without LNM. Patients with missing values for any variable included in the multivariable model were excluded. No imputation was performed. ^**^P<0.01, ^*^P<0.05.

## Discussion

Previous studies have established that traditional clinicopathological factors, including histological grade, pathological subtype, FIGO stage, depth of myometrial invasion, and LVSI, were intimately associated with LNM in endometrial carcinoma (11). However, relying exclusively on these conventional parameters remains insufficient to meet the contemporary clinical demand for precise risk stratification. In the present study, our univariate analysis revealed that menopausal status, histological grade, pathological subtype, depth of myometrial invasion, and LVSI were all significantly associated with LNM. Within the multivariate model, LVSI ultimately emerged as the sole traditional clinicopathological parameter serving as an independent associated factor for LNM. LVSI is defined by the presence of tumor cells within the lumen of lymphatic vessels or capillaries. It represents the crucial, obligatory initial step for both regional lymphatic spread and distant hematogenous dissemination. Extensive research has consistently corroborated that LVSI positivity is a predictor of lymphatic involvement and adverse oncological outcomes in endometrial carcinoma (6, 12). Our findings strongly reaffirm the indispensable, core role of LVSI within the clinical evaluation paradigm for assessing LNM risk.

In the present study, the univariate analysis demonstrated that the expression levels of ER, PR, p53, Ki67, and HER2 were significantly associated with LNM in endometrial carcinoma. However, subsequent multivariate logistic regression analysis confirmed that high expression of HER2 and p53, were definitive independent associated factors for LNM. Regarding molecular biomarkers, HER2 staining level demonstrated the largest estimated association with LNM in the current study. As a classic proto-oncogene-encoded protein, HER2 actively drives tumor progression by promoting cellular proliferation, migration, and invasion (13). Our finding that elevated HER2 expression as an independent associated factor for LNM is highly consistent with existing literature (14, 15), which closely associates HER2 positivity with high-risk clinicopathological features and adverse clinical outcomes in endometrial carcinoma. Beyond its substantial value in preoperative risk assessment, HER2 status provides a critical basis for therapeutic stratification. Previous studies have demonstrated that the addition of trastuzumab to standard chemotherapy significantly improves clinical outcomes in patients with HER2-overexpressing uterine serous carcinoma (16). Furthermore, with the continuous evolution of systemic therapies for advanced or recurrent endometrial carcinoma, the pivotal role of molecular biomarkers in guiding individualized treatments has garnered unprecedented attention (17). For instance, it is now well-established that high microsatellite instability (MSI-H) or mismatch repair-deficient (dMMR) status strongly correlates with therapeutic benefit from PD-1 inhibitors (18). However, these findings require careful interpretation because HER2 in the present study was assessed using a historical semi-quantitative immunohistochemical classification based on the proportion of positively stained tumor cells (negative, +, ++, and +++), rather than contemporary endometrial cancer-specific HER2 scoring criteria. The study also did not retrospectively reclassify HER2 according to current disease-specific criteria or systematically assess HER2 gene amplification. Therefore, the observed association should be interpreted as an association between historical semi-quantitative HER2 staining level and LNM, rather than as evidence of contemporary HER2-positive disease, HER2 amplification, or eligibility for HER2-directed therapy. Future studies using standardized, contemporary endometrial cancer-specific HER2 assessment are warranted to determine whether this association remains evident under current biomarker definitions.

The P53 gene encodes a critical tumor suppressor intimately involved in cell cycle regulation and DNA damage repair. Consequently, abnormal p53 expression typically signifies profound cell cycle dysregulation and an augmented propensity for malignant progression. Consistent with prior research demonstrating a correlation between p53 alterations and poor prognosis, P53-mutated (abnormal p53, p53abn) endometrial carcinomas are classically characterized by heightened invasiveness and inferior clinical outcomes (19).In the present study, higher p53 immunohistochemical expression was independently associated with LNM. The interpretation of p53 expression in the present study requires particular caution. Contemporary molecular classification of endometrial carcinoma uses p53 immunohistochemistry as a surrogate for TP53 status, with p53-abnormal classification based on characteristic staining patterns rather than simply the proportion of p53-positive tumor cells. Current guidelines recognize aberrant p53 patterns such as diffuse overexpression, complete absence (null pattern), or cytoplasmic staining. In contrast, our retrospective cohort used a historical semi-quantitative scoring system based on the proportion of p53-positive tumor cells. Therefore, the p53 results in this study should be interpreted as associations between the semi-quantitative p53 staining level and LNM, rather than as evidence of p53-abnormal molecular classification or TP53 mutation status. Therefore, further studies incorporating molecular testing are required to determine whether the observed association reflects specific TP53 genetic alterations.

Notably, although elevated Ki67 expression, along with the loss of ER and PR, demonstrated significant associations with LNM in the univariate analysis, they failed to emerge as independent predictive factors within the multivariate model. Furthermore, other biomarkers such as ERCC1 showed no significant correlation with LNM in our study. These observations suggest that the absence of hormone receptors and the expression of drug-resistance molecules may primarily reflect the tumor’s responsiveness to endocrine or systemic therapies and dictate overall survival outcomes, rather than directly driving the biological cascade of lymphatic dissemination.

Despite these promising findings, several limitations should be acknowledged. First, the single-center, retrospective design may have introduced selection bias and limited the generalizability of the findings center. Second, because the cohort spanned more than 10 years, different antibody reagents and/or clones were used for immunohistochemical staining according to the pathology laboratory protocols in place at the time. Although the IHC results were recorded using the same semiquantitative categories, inter-assay and inter-reagent variability cannot be completely excluded. In particular, p53 was assessed using a historical semiquantitative approach rather than contemporary pattern-based classification. Therefore, the associations observed for HER2 and p53 should be interpreted as findings based on historical IHC expression measurements rather than definitive molecular or p53-abnormal classifications. Future studies using standardized antibody platforms and contemporary endometrial cancer-specific scoring criteria are warranted. Third, although the multivariable analysis identified several factors independently associated with LNM, only 36 LNM events were included in the complete-case multivariable model. Given the limited number of events relative to the number of covariates, the possibility of model overfitting cannot be excluded. Therefore, the multivariable findings should be considered exploratory and require validation in larger, multicenter cohorts. Fourth, the current analysis did not integrate the comprehensive molecular classification framework proposed by The Cancer Genome Atlas (TCGA), such as the evaluation of POLE mutational status. Consequently, the independent clinical predictive value of HER2 and p53 requires further corroboration within the context of standardized molecular subtyping. In addition, the historical Bokhman Type I/Type II classification does not adequately capture the molecular heterogeneity of endometrial cancer and should not be considered equivalent to contemporary molecular classification. These factors may have limited our ability to fully characterize the relationship between HER2, p53, LVSI, and LNM across molecularly distinct tumor subgroups. Future multicenter studies with standardized IHC assessment and integrated molecular classification are needed to validate these findings and to further clarify whether HER2, p53, and LVSI may provide clinically useful information for risk stratification and surgical decision-making in patients with endometrial cancer.

In summary, our analysis confirms that higher HER2 and p53 expression and the presence of LVSI were independently associated with LNM. Because these biomarkers were assessed in postoperative surgical specimens, the present findings do not establish their value for preoperative or intraoperative prediction. Further studies are needed to determine whether HER2 and p53 assessment in preoperative biopsy specimens can provide clinically useful prediction of LNM.

## Data Availability

The original contributions presented in the study are included in the article/supplementary material. Further inquiries can be directed to the corresponding author.
